# Supplementary material for: A Short Corticosteroid Course Reduces Symptoms and Immunological Alterations Underlying Long-COVID
Source: Biomedicines. 2021 Oct 26;9(11):1540. doi: 10.3390/biomedicines9111540 (PMC8614904; doi:10.3390/biomedicines9111540)
Supplement: Supplementary file 1 [file biomedicines-09-01540-s001.zip › Supplementary Figures.pdf]

Supplementary Materials

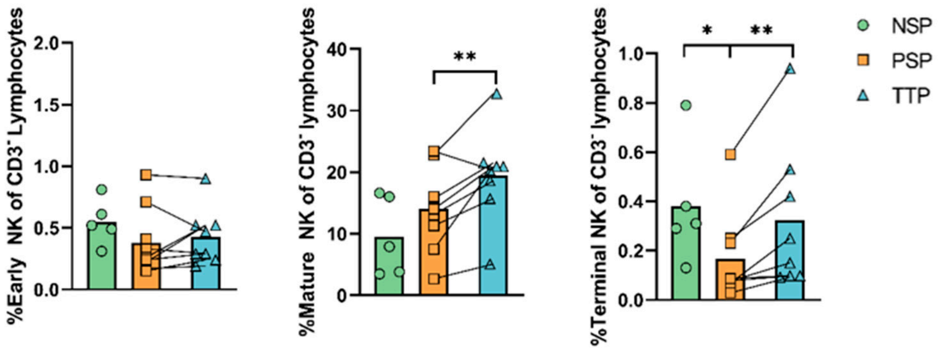

Figure S1. NK cell subpopulations in NSP, PSP and TTP. *, $p < 0.05$; **, $p < 0.01$.

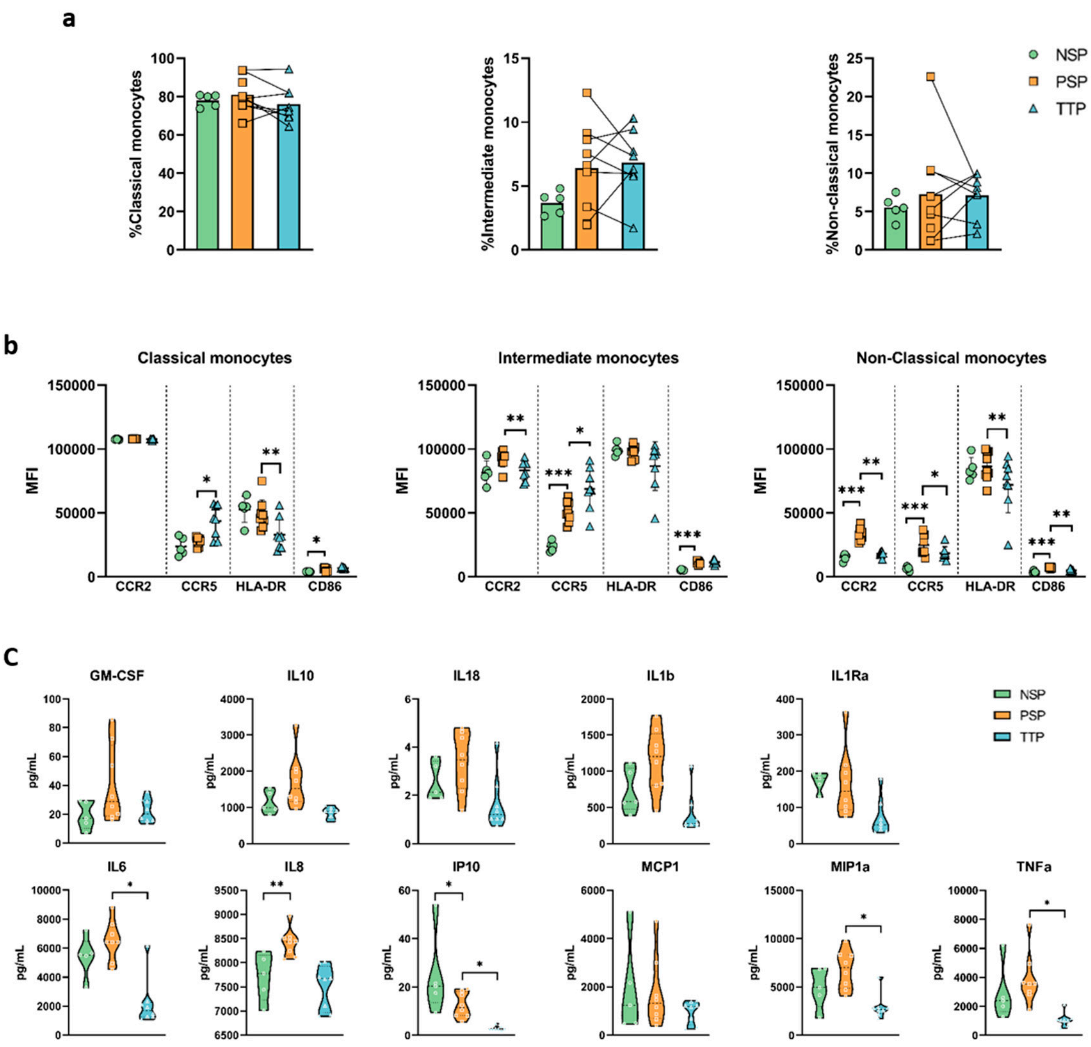

Figure S2. Higher expression of chemotactic and co-stimulatory molecules on monocytes from PSP. (**a**) Comparison of classical, intermediate and non-classical monocytes between NSP, PSP and TTP. (**b**) Expression of CCR2, CCR5, HLA-DR and CD86 on classical, intermediate and non-classical monocytes from NSP, PSP and TTP. (**c**) Secretion of GM-CSF, IL10, IL18, IL1b, IL1Ra, IL6, IL8, IP10, MCP1, MIP1a and TNFa upon LPS in vitro stimulation of monocytes isolated from NSP, PSP and TTP. *, $p < 0.05$; **, $p < 0.01$; ***, $p < 0.001$.

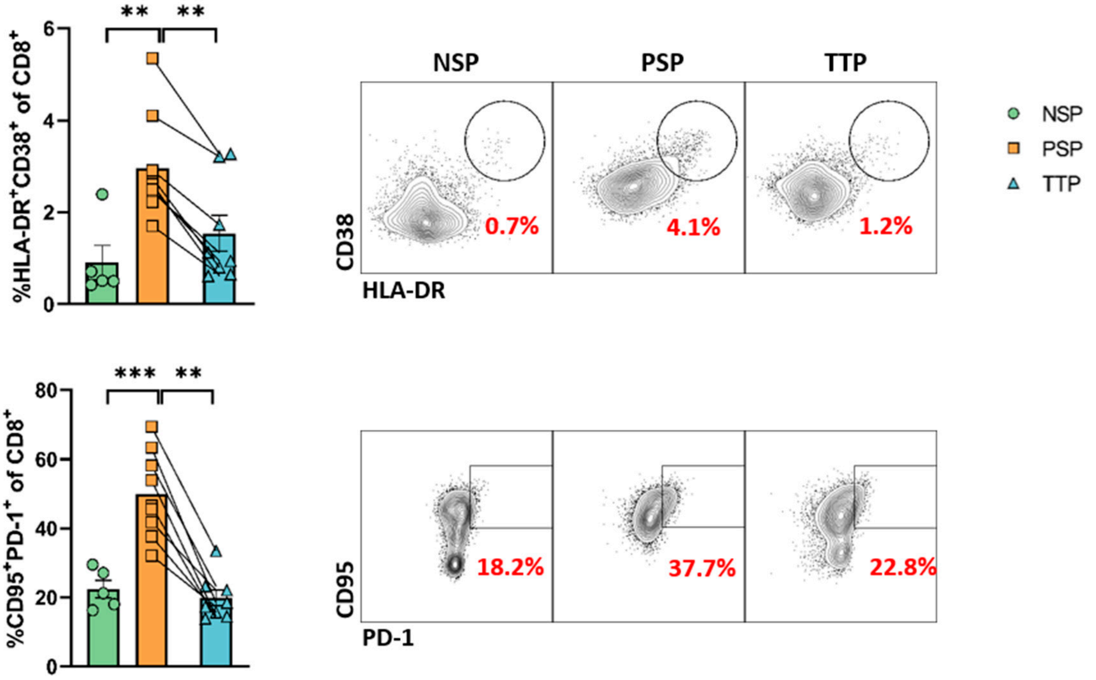

Figure S3. Expression of activation and senescence markers on CD8 T cells. **, $p < 0.01$; ***, $p < 0.001$.

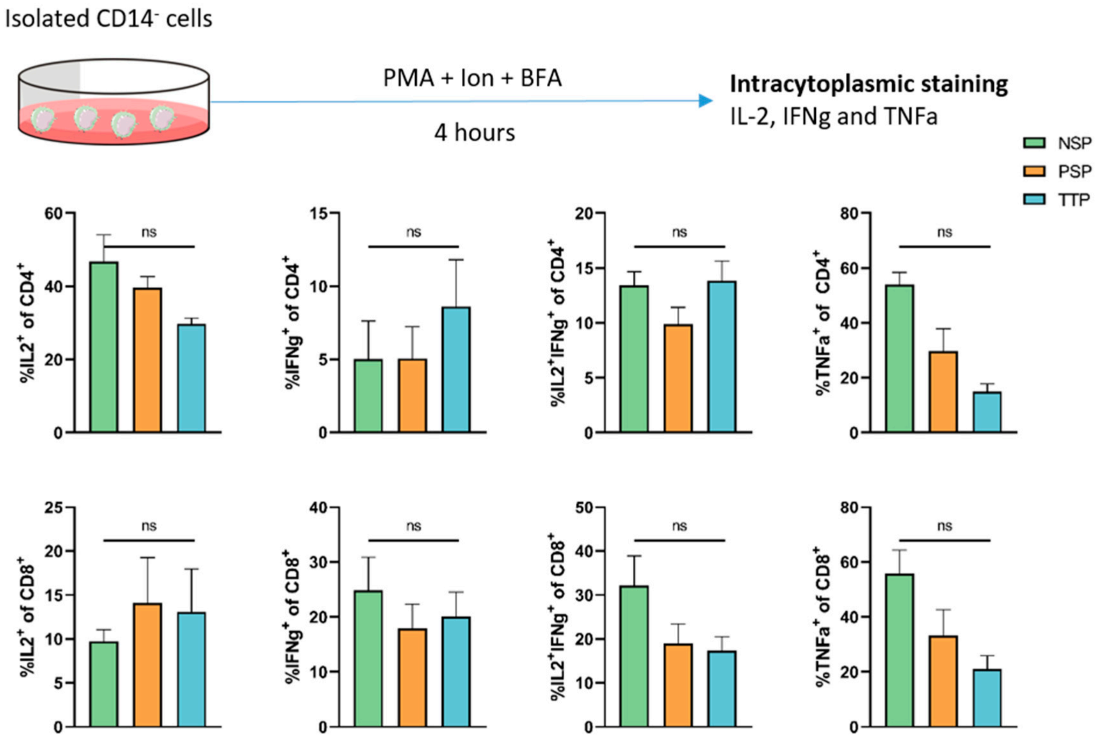

Figure S4. Intracytoplasmic pro-inflammatory cytokine detection in CD4+ and CD8+ T cells upon in vitro stimulation.

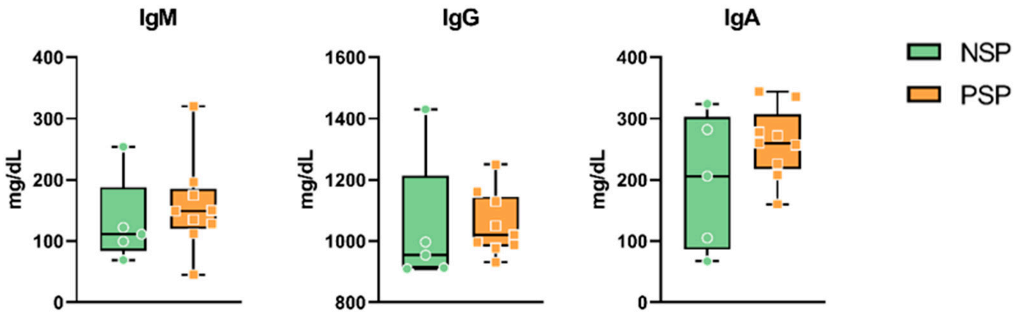

Figure S5. Immunoglobulin M, G and A levels in plasma from NSP and PSP.

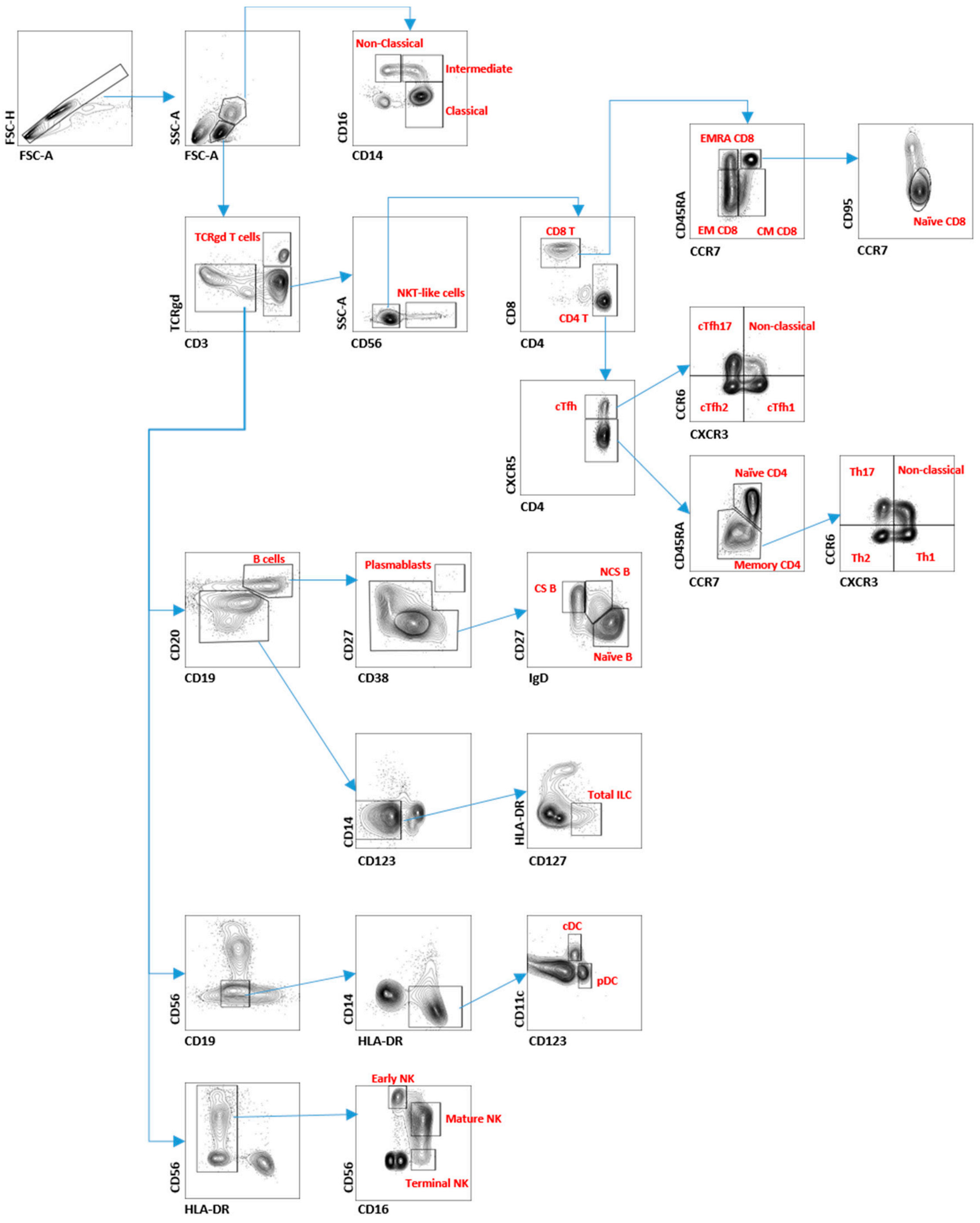

Figure S6. Flow cytometry gating strategy.
