# Supplementary material for: A Short Corticosteroid Course Reduces Symptoms and Immunological Alterations Underlying Long-COVID
Source: Biomedicines. 2021 Oct 26;9(11):1540. doi: 10.3390/biomedicines9111540 (PMC8614904; doi:10.3390/biomedicines9111540)
Supplement: Supplementary file 1 [file biomedicines-09-01540-s001.zip › Supplementary Tables.pdf]

Supplementary Materials

Table S1. Characteristics of post-COVID-19 patients.

Patient	Group	Sex	Age	Hospitalization	Months from acute COVID-19	Arthralgia	Myalgia	Dyspnea	Asthenia	Paresthesia	Dizziness	Anxiety	Insomnia	Headache	Brain fog	Anosmia	Dysgeusia
Patient 1	NSP	Male	25	No	6	No	No	No	No	No	No	No	No	No	No	No	No
Patient 2	NSP	Female	26	No	8	No	No	No	No	No	No	No	No	No	No	No	No
Patient 3	NSP	Male	62	No	6	No	No	No	No	No	No	No	No	No	No	No	No
Patient 4	NSP	Female	56	Yes	13	No	No	No	No	No	No	No	No	No	No	No	No
Patient 5	NSP	Female	52	No	3	No	No	No	No	No	No	No	No	No	No	No	No
Patient 6	PSP	Female	61	No	13	Yes	Yes	Yes	Yes	Yes	No	Yes	Yes	Yes	No	No	No
Patient 6	TTP					Complete R.	Complete R.	Complete R.	Complete R.	Complete R.	No	Complete R.	Complete R.	Complete R.	No	No	No
Patient 7	PSP	Female	49	No	3	Yes	Yes	No	No	Yes	No	No	No	No	No	No	No
Patient 7	TTP					Partial R.	Partial R.	No	No	Complete R.	No	No	No	No	No	No	No
Patient 8	PSP	Male	56	Yes	13	Yes	Yes	Yes	Yes	Yes	Yes	Yes	Yes	No	Yes	Yes	No
Patient 8	TTP					No R.	No R.	Partial R.	No R.	No R.	No R.	No R.	No R.	No	No R.	No R.	No
Patient 9	PSP	Female	43	No	8	Yes	Yes	Yes	Yes	Yes	Yes	No	Yes	Yes	Yes	Yes	Yes
Patient 9	TTP					Partial R.	Partial R.	No R.	Partial R.	No R.	No R.	No	No R.	No R.	No R.	No R.	No R.
Patient 10	PSP	Female	44	No	12	Yes	Yes	Yes	Yes	Yes	Yes	No	No	Yes	Yes	No	No
Patient 10	TTP					Partial R.	Partial R.	No R.	No R.	No R.	No R.	No	No	No R.	No R.	No	No
Patient 11	PSP	Female	31	No	13	Yes	Yes	Yes	Yes	No	Yes	No	No	Yes	No	No	No
Patient 11	TTP					Complete R.	Partial R.	No R.	No R.	No	No R.	No	No	No R.	No	No	No
Patient 12	PSP	Female	30	No	7	Yes	Yes	Yes	Yes	Yes	No	Yes	Yes	No	No	No	No
Patient 12	TTP					Partial R.	Partial R.	No R.	Partial R.	No R.	No	No R.	No R.	No	No	No	No
Patient 13	PSP	Female	33	Yes	12	No	Yes	Yes	Yes	Yes	Yes	Yes	Yes	Yes	Yes	No	No
Patient 13	TTP					No	No R.	No R.	Partial R.	No R.	Partial R.	No R.	Partial R.	Partial R.	No R.	No	No
Patient 14	PSP	Female	35	No	3	No	No	Yes	Yes	No	No	Yes	No	No	No	Yes	Yes

NSP: no symptoms patients; PSP: persistent symptoms patients; TTP: treated patients; R.: recovery.

Table S2. Hemogram of each individual.

	NSP					PSP									TTP								
**Patients**	**P1**	**P2**	**P3**	**P4**	**P5**	**P6**	**P7**	**P8**	**P9**	**P10**	**P11**	**P12**	**P13**	**P14**	**P6**	**P7**	**P8**	**P9**	**P10**	**P11**	**P12**	**P13**	**Normal values**
Platelets	277	261	246	232	190	209	182	219	246	214	286	370	212	350	223	203	234	238	223	264	389	193	140 - 450
Neutrophils (10^6 cells/uL)	3.2	2.6	3.7	4.3	3.2	3.3	4.5	4.1	2.3	2.6	2.2	5.6	3.2	4.8	6	11	5.2	6.3	5.6	7	10.2	7.6	1.8 - 7.6
Neutrophils (%)	52.4	49.3	61.5	59.8	59.1	49.5	66.2	62.1	56.5	49.8	37.8	62.1	52.2	56.5	83.9	92.9	82.7	85.9	83.7	85.3	88.2	78	41.0 - 72.7
Lymphocytes (10^6 cells/uL)	2.3	2.2	1.8	2.1	1.8	2.8	1.7	1.7	1.4	2.1	2.7	2.3	2.3	3.1	1.1	0.7	0.9	0.9	1	1.1	1.1	1.6	1.2 - 4.2
Lymphocytes (%)	38.2	42.5	29.5	29.4	32.5	42	24.5	25.9	33.4	40.3	46.8	25.3	37.4	36.6	15.1	5.9	14.2	12.3	14.2	13.7	9.7	16.4	20.1 - 49.0
Monocytes (10^6 cells/uL)	0.4	0.4	0.3	0.6	0.4	0.4	0.6	0.6	0.4	0.4	0.4	0.8	0.6	0.5	0.1	0.1	0.2	0.1	0.1	0.1	0.2	0.5	0.2 - 0.9
Monocytes (%)	6.9	6.9	5.3	7.9	6.8	6.6	8.1	8.5	8.9	7.5	6.9	8.6	10.3	6.1	0.9	1.1	2.6	1.6	1.8	0.8	1.9	5.5	3.4 - 10.6
Eosinophils (10^6 cells/uL)	0.1	0	0.2	0.1	0.1	0.1	0.1	0.1	0	0.1	0.5	0.3	0	0	0	0	0	0	0	0	0	0	0.0 - 0.5
Eosinophils (%)	2.1	0.9	3.2	2.1	1	1.4	0.8	2.3	0.5	1.7	7.7	3.7	0	0.4	0	0	0.1	0	0.1	0	0	0	0.3 - 5.6

Basophils (10^6 cells/uL)	0	0	0	0.1	0	0	0	0.1	0	0	0	0	0	0	0	0	0	0	0	0	0	0	0.0 - 0.2
Basophils (%)	0.4	0.4	0.5	0.8	0.6	0.5	0.4	1.2	0.7	0.7	0.8	0.3	0.1	0.4	0.1	0.1	0.4	0.2	0.2	0.2	0.2	0.1	0.2 - 2.1

P: patient; NSP: no symptoms patients; PSP: persistent symptoms patients; TTP: treated patients.

Table S3. Autoantibodies.

			NSP							PSP				
**Patients**	**P1**	**P2**	**P3**	**P4**	**P5**	**P6**	**P7**	**P8**	**P9**	**P10**	**P11**	**P12**	**P13**	**P14**
**APLS**														
IgA aB2	Negative	Negative	Negative	Negative	Negative	Negative	Positive	Negative	Negative	Negative	Negative	Negative	Negative	Negative
IgG aB2	Negative	Negative	Negative	Negative	Negative	Negative	Negative	Negative	Negative	Negative	Negative	Negative	Negative	Negative
IgM aB2	Negative	Negative	Negative	Negative	Negative	Negative	Negative	Low positive	Negative	Negative	Negative	Negative	Negative	Negative
IgG aCL	Negative	Negative	Negative	Negative	Negative	Negative	Negative	Negative	Negative	Negative	Negative	Negative	Negative	Negative
IgM aCL	Negative	Negative	Negative	Negative	Negative	Negative	Negative	Low positive	Negative	Negative	Negative	Negative	Negative	Negative
**ANCA**														
MPO	Negative	Negative	Negative	Negative	Negative	Negative	Negative	Negative	Negative	Negative	Negative	Negative	Negative	Negative
PR3	Negative	Negative	Negative	Negative	Negative	Negative	Negative	Negative	Negative	Negative	Negative	Negative	Negative	Negative
**ANA**														
dsDNA	Negative	Negative	Negative	Negative	Negative	Negative	Negative	Positive	Negative	Negative	Positive	Negative	Negative	Negative
Chrom	Negative	Negative	Negative	Negative	Negative	Negative	Negative	Negative	Negative	Negative	Negative	Negative	Negative	Negative
Ribo P	Negative	Negative	Negative	Negative	Negative	Negative	Negative	Negative	Negative	Negative	Negative	Negative	Negative	Negative
SS-A	Negative	Negative	Negative	Negative	Negative	Negative	Negative	Negative	Negative	Negative	Negative	Negative	Negative	Negative
SS-A52	Negative	Negative	Negative	Negative	Negative	Negative	Negative	Negative	Negative	Negative	Negative	Negative	Negative	Negative
SS-B	Negative	Negative	Negative	Negative	Negative	Negative	Negative	Negative	Negative	Negative	Negative	Negative	Negative	Negative
Cent B	Negative	Negative	Negative	Negative	Negative	Negative	Negative	Negative	Negative	Negative	Negative	Negative	Negative	Negative
Sm	Negative	Negative	Negative	Negative	Negative	Negative	Negative	Negative	Negative	Negative	Negative	Negative	Negative	Negative
SmRNP	Negative	Negative	Negative	Negative	Negative	Negative	Negative	Negative	Negative	Negative	Negative	Negative	Negative	Negative
RNP 68	Negative	Negative	Negative	Negative	Negative	Negative	Negative	Negative	Negative	Negative	Negative	Negative	Negative	Negative
RNP A	Negative	Negative	Negative	Negative	Negative	Positive	Negative	Negative	Negative	Negative	Negative	Negative	Negative	Negative
Scl-70	Negative	Negative	Negative	Negative	Negative	Negative	Negative	Negative	Negative	Negative	Negative	Negative	Negative	Negative
Jo-1	Negative	Negative	Negative	Negative	Negative	Negative	Negative	Negative	Negative	Negative	Negative	Negative	Negative	Negative
**Myositis**														
PL-7	Negative	Negative	Negative	Negative	Negative	Negative	Negative	Negative	Negative	Negative	Negative	Negative	Negative	Negative
PL-12	Negative	Negative	Negative	Negative	Negative	Negative	Negative	Negative	Negative	Negative	Negative	Negative	Negative	Negative
EJ	Negative	Negative	Negative	Negative	Negative	Negative	Negative	Negative	Negative	Negative	Negative	Negative	Negative	Negative
SRP	Negative	Negative	Negative	Negative	Negative	Negative	Negative	Negative	Negative	Negative	Negative	Negative	Negative	Negative
MI-2	Negative	Negative	Negative	Negative	Negative	Negative	Negative	Negative	Negative	Negative	Negative	Negative	Negative	Negative
MDA-5	Negative	Negative	Negative	Negative	Negative	Negative	Negative	Negative	Negative	Negative	Negative	Negative	Negative	Negative
TIF1-gamma	Negative	Negative	Negative	Negative	Negative	Negative	Negative	Negative	Negative	Negative	Negative	Negative	Negative	Negative
Ku	Negative	Negative	Negative	Negative	Negative	Negative	Negative	Negative	Negative	Negative	Negative	Negative	Negative	Negative
PM-Scl 100	Negative	Negative	Negative	Negative	Negative	Negative	Negative	Negative	Negative	Negative	Negative	Negative	Negative	Negative
Scl-70	Negative	Negative	Negative	Negative	Negative	Negative	Negative	Negative	Negative	Negative	Negative	Negative	Negative	Negative
SSA/Ro52kDa	Negative	Negative	Negative	Negative	Negative	Negative	Negative	Negative	Negative	Negative	Negative	Negative	Negative	Negative

**Neuronal**														
GAD65	Negative	Negative	Negative	Negative	Negative	Negative	Negative	Negative	Negative	Negative	Negative	Negative	Negative	Negative
SOX1	Negative	Negative	Negative	Negative	Negative	Negative	Negative	Negative	Negative	Negative	Negative	Negative	Negative	Negative
Ma2	Negative	Negative	Negative	Negative	Negative	Negative	Negative	Negative	Negative	Negative	Negative	Negative	Negative	Negative
Ma1	Negative	Negative	Negative	Negative	Negative	Negative	Negative	Negative	Negative	Negative	Negative	Negative	Negative	Negative
Amphiphysin	Negative	Negative	Negative	Negative	Negative	Negative	Negative	Negative	Negative	Negative	Negative	Negative	Negative	Negative
CV 2 (CRMP5)	Negative	Negative	Negative	Negative	Negative	Negative	Negative	Negative	Negative	Negative	Negative	Negative	Negative	Negative
Ri	Negative	Negative	Negative	Negative	Negative	Negative	Negative	Negative	Negative	Negative	Negative	Negative	Negative	Negative
Yo	Negative	Negative	Negative	Negative	Negative	Negative	Negative	Negative	Negative	Negative	Negative	Negative	Negative	Negative
HuD	Negative	Negative	Negative	Negative	Negative	Negative	Negative	Negative	Negative	Negative	Negative	Negative	Negative	Negative

NSP: no symptoms patients; PSP: persistent symptoms patients; TTP: treated patients.
